# The Potential Use of Electrochemotherapy in the Treatment of Uveal Melanoma: In Vitro Results in 3D Tumor Cultures and In Vivo Results in a Chick Embryo Model

**DOI:** 10.3390/cancers11091344

**Published:** 2019-09-11

**Authors:** Miltiadis Fiorentzis, Arne Viestenz, Udo Siebolts, Berthold Seitz, Sarah E. Coupland, Joana Heinzelmann

**Affiliations:** 1 Department of Ophthalmology, University Hospital Halle (Saale), Martin-Luther University Halle-Wittenberg, Ernst-Grube-Str. 40, 06120 Halle (Saale), Germany; arne.viestenz@uk-halle.de (A.V.); joana.heinzelmann@uk-halle.de (J.H.); 2 Department of Pathology, University Hospital Halle (Saale), Martin-Luther University Halle-Wittenberg, Magdeburger Str. 14, 06112 Halle (Saale), Germany; udo.siebolts@uk-halle.de; 3 Department of Ophthalmology, Saarland University Medical Center, Kirrberger Str. 100, 66421 Homburg/Saar, Germany; berthold.seitz@uks.eu; 4 Liverpool Ocular Oncology Research Group, Department of Molecular and Clinical Cancer Medicine, Institute of Translational Medicine, University of Liverpool, West Derby Street, Liverpool L7 8TX, UK; S.E.Coupland@liverpool.ac.uk; 5 Liverpool Clinical Laboratories, Royal Liverpool University Hospital, Liverpool L69 3GA, UK

**Keywords:** electrochemotherapy, uveal melanoma, tumor spheroids, chick embryo chorioallantoic membrane, treatment, bleomycin, electroporation, in vitro, in vivo

## Abstract

Uveal melanoma (UM) is the most common primary intraocular tumor that arises from neoplastic melanocytes in the choroid, iris, and ciliary body. Electrochemotherapy (ECT) has been successfully established for the treatment of skin and soft tissue metastatic lesions, deep-seated tumors of the liver, bone metastases, and unresectable pancreas lesions. The aim of this study was to evaluate the effect of ECT in vitro in 3D spheroid culture systems in primary and metastatic UM cell lines. We also investigated the chick embryo chorioallantoic membrane (CAM) as an in vivo model system for the growth and treatment of UM tumors using ECT. The cytotoxic effect of ECT in 3D spheroids was analyzed seven days following treatment by assessment of the size and MTT [(3-(4,5-dimethylthiazol-2-yl)-2,5-diphenyltetrazolium bromide) tetrazolium reduction] assay. The cytotoxicity of ECT after intratumoral or intraarterial administration was evaluated histologically. In vitro and in vivo ECT caused a significant reduction in tumor size and viability compared to electroporation or chemotherapy in both sections of our study. The current results underline the effectiveness of ECT in the treatment of UM and prepare the way for further investigation of its potential application in UM.

## 1. Introduction

Uveal melanoma (UM) is the most common primary intraocular tumor in adults, with an incidence of 5.1 cases per million annually in the USA and between 4 and 7 per million per year in Europe [1]. Most of the tumors arise in the choroid (85%), whereas the remaining cases occur in the ciliary body and the iris [2]. A higher malignancy is observed in melanomas of the ciliary body, which are detected later and metastasize more frequently. Most UM are confined to the globe on diagnosis; however, large or diffuse lesions may show extrascleral extension [3]. As a result of the increased vascularity of the uveal tissue, particularly the choroid, UM cells can spread to distant organs via the bloodstream [3]. An extraocular extension may occur along the optic nerve and the lumen of vortex veins [1]. Lymphatic spread is extremely rare and typically occurs only when there is anterior extension into the conjunctiva [3]. The liver (93%), lungs (24%), and bones (16%) are the most common metastasizing sites of UM [1,2,3].

The development of UM is associated with the presence of related ocular or cutaneous melanocytic lesions, fair complexion and a light iris [4]. The molecular pathogenesis of UM is characterized by specific chromosome alterations and gene mutations [5]. The most common abnormalities include loss of 1p, 3, 6q, and 8p as well as a gain of 1q, 6p, and 8q chromosomes. Monosomy 3 (i.e., the loss of one copy of chromosome 3) occurs in almost half of UM cases, constituting an important prognostic marker for the clinical outcome of UM [5]. Furthermore, UM is characterized in ~80% of cases by mutually exclusive initiating mutations in guanine nucleotide binding protein G(q) subunit alpha (*GNAQ*) and quinine nucleotide binding protein subunit alpha 11 (*GNA11*) [5]. Other genes involved in UM development include *CYSLTR2*, and *PLCB4*, while those involved in UM progression/metastasis include *BAP1*, *SF3B1*, *EIF1AX*, and *SRSF2*. There are some mutations detected at a low frequency (e.g., *CSDM1*, *TTC28*, *TP53BP1*, *DLK2*, and *KTN1*), where the clinical significance is currently unknown [6,7].

The first-line therapeutic options for UM include radiotherapy (brachytherapy and proton beam radiotherapy), phototherapy (photocoagulation, transpupillary thermal therapy, and photodynamic therapy), and surgery (local resection, enucleation, and exenteration) [1]. The selected therapeutic approach depends on the size and location of the tumor as well as on the individual decision of the patient [8]. Immunotherapy, chemotherapy, and molecular targeted therapy are used as adjuvant treatments for metastatic UM [8].

Electrochemotherapy (ECT) and its effectiveness have been established in the treatment of skin and superficial soft tissue metastatic lesions, and recently have been described for the application in deep-seated tumors of the liver, bone metastases, and unresectable pancreas cancer [9,10,11]. In addition, promising results have been achieved for the treatment of periocular basal cell carcinomas [12]. The role of ECT in the management of ocular melanomas—i.e., UM and conjunctival melanoma—has yet to be clarified.

Electroporation (EP) increases the permeability of the cell membrane after the application of short electric pulses on both sides of the membrane, resulting in the formation of transient pores, allowing for the delivery of large hydrophilic molecules to the cytosol [10,13]. The pulse amplitude and number of pulses can lead to a reversible EP with membrane permeability recovery, or to an irreversible effect. The reversible EP can be performed to introduce genetic material or drugs into cells without affecting the cell viability [10]. The combination of EP and drug injection is referred to as ECT and can enhance the cytotoxic effect of the applied agent. Bleomycin and cisplatin show the highest potential cytotoxic effect, in combination with EP [10]. ECT is a local and nonthermal tumor ablation modality that is currently used in clinical practice to treat cutaneous and subcutaneous nodules in patients with progressive disease of different malignances, e.g., soft tissue sarcomas and carcinomas, cutaneous melanoma as well as colorectal liver metastases, tumor nodules in the proximity of important structures like vessels and nerves, and tumors not amenable to excision or radiofrequency ablation [14]. There is currently little knowledge regarding the effect of ECT in ocular melanoma [15]. Due to the reduced drug doses needed for the ECT, most of the observed adverse effects are local and transient, including local pain, erythema, edema, and muscle contractions [9].

In vitro 3D culture systems have been developed to incorporate in vivo growth conditions of tumor masses, and to better preserve the biological characteristics and microenvironment of tumors than conventional monolayer cultures [16]. Tumor-derived spheroids are unique and are used for the reinforcement of cells with stem-cell-related characteristics. These grow as floating spheres and constitute an assessment system to analyze the cancer stem-cell-related characteristics of solid tumors in vitro [17]. The ethical and technical limitations of animal models in oncology research emphasize the value and necessity of further in vivo models, such as the chick embryo [18,19]. Such in vitro and in vivo preclinical models reproduce the molecular and cellular processes of the UM. The chick embryo chorioallantoic membrane (CAM) is a highly vascularized extraembryonic membrane and provides a technically simple method of visualization and investigation for complex biological systems [18]. The lack of an immune system in the early stages has established the chick model as a decisive model in cancer research [18,19,20].

The aim of this study was to evaluate the effect of bleomycin in five primary and metastatic UM spheroid culture systems, when combined with EP or used alone. Subsequently, we tested the CAM as an in vivo assay to develop and treat UM nodular masses.

## 2. Results

### 2.1. ECT of 3D Spheroids

**Figure 1:**
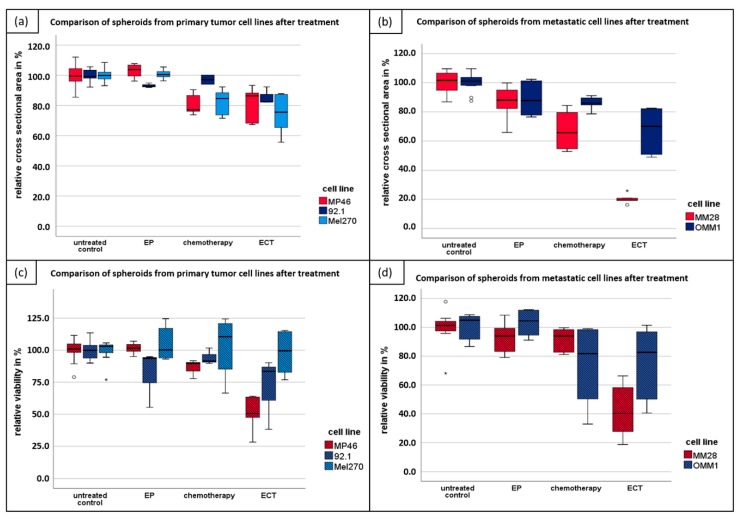
Electrochemotherapy (ECT, 750V/cm after application of 2.5 µg/mL bleomycin) caused stronger cytotoxic effects in spheroids of UM cell lines compared to electroporation (EP) or chemotherapy using bleomycin (2.5 µg/mL) alone. The cytotoxic effect was measured by calculating both the cross-sectional area and MTT assay as a percentage of the untreated control seven days after treatment. Box plots show (**a**) the mean cross-sectional area of spheroids from primary UM cell lines; (**b**) the mean cross-sectional area of spheroids from metastatic UM cell lines; (**c**) the mean viability of spheroids from primary UM cell lines; (**d**) the mean viability of spheroids from metastatic UM cell lines. Uveal melanoma cell lines with monosomy 3 and missing BAP1 expression are labeled in red; cell lines without LOH of chromosome 3 and verified BAP1 expression are marked in blue. Outliers with values between 1.5 and 3 times of interquartile range are marked by an “ο” and extremes with values more than 3 times of interquartile range are marked by an asterisk “*”.

The spheroid growth of different UM cell lines of primary and metastatic origin was calculated in the cross-sectional area of the spheroids after seven days of treatment. In four of five cell lines, the most significant reduction of size after ECT compared to untreated spheroids (control group) was detected in primary tumor cell lines and metastatic cell lines after treatment with 750 V/cm after application of 2.5 µg/mL bleomycin (ECT) (Figure 1a,b).

**Figure 2:**
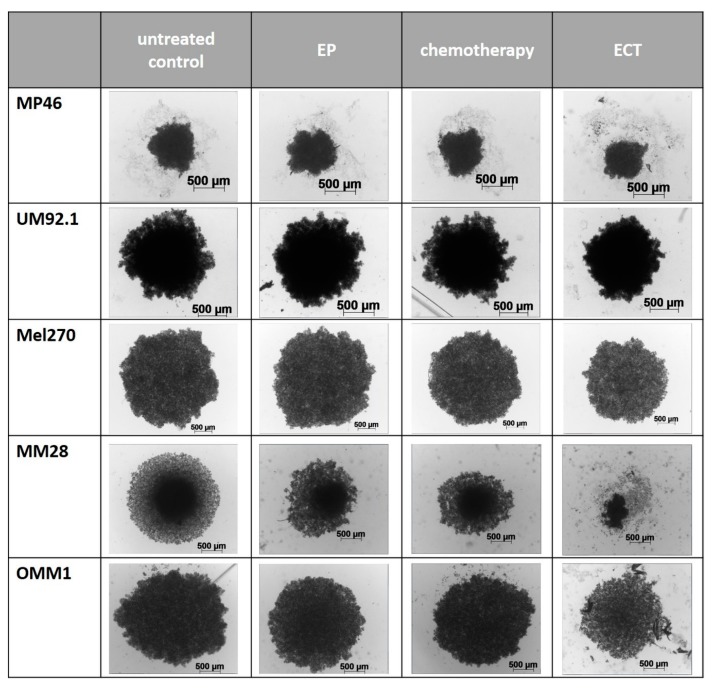
Representative images of spheroids from primary UM cell lines (MP46, 92.1, Mel270) and metastatic UM cell lines (MM28, OMM1) after electrochemotherapy (ECT) compared to electroporation alone (EP), chemotherapy using bleomycin (2.5 µg/mL) alone and untreated spheroids at day seven following treatment. Scale bar = 500 µm.

Chemotherapy alone and EP alone showed a nonsignificant reduction of spheroid growth. The cytotoxic effect of all tested therapeutic modalities was higher in metastatic than in primary tumor cell lines. The mean difference compared to the control group was 56.2% and 19.4%, respectively. Morphological changes of spheroids at day seven following ECT were more prominent in the metastatic cell lines and resulted in a loss of sphericity and loose cells around the spheroid (Figure 2). The average growth differences are shown in Table 1.

The UM cell lines formed not spherical but oblate spheroids, with a loose association of cells. To validate the microscopic observations of spheroid size, a viability assay was performed using MTT assay seven days following treatment (Figure 1c,d). Chemotherapy alone and EP alone showed only a limited effect on the spheroid’s morphology and size. In all cell lines, apart from the Mel270, the highest cytotoxicity was observed after ECT. The average differences in viability following EP alone, chemotherapy alone, and ECT are shown in Table 2.

### 2.2. In Vivo Effects of ECT on Uveal Melanoma Cells Using Chick Embryo Chorioallantoic Membrane (CAM) Assay

The 92.1 cell line is a highly pigmented UM cell line, making it suitable for an in vivo CAM assay, since the cell‒Matrigel grafts can be easily observed for a long period of time without artificial labeling of the cells. We utilized the ‘in ovo’ CAM assay to analyze in vivo the cytotoxic effect of ECT on UM cells based on cell line 92.1. The survival rate of the embryos was 52% on embryonic day 16 (E16).

**Figure 3:**
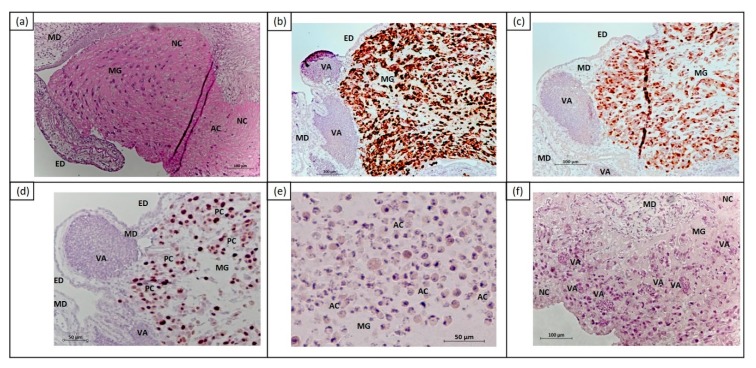
FFPE sections of CAM including human 92.1 UM cells for analysis of cytotoxic effects of electrochemotherapy at E16 FFPE sections. (**a**) Matrigel grafts in CAM layer were visualized (in red) using PAS staining; (**b**) 92.1 cells in CAM layer were immunohistochemically stained with antihuman melanosome; (**c**) 92.1 cells in CAM layer were immunohistochemically stained with MelanA; (**d**) 92.1 cells in CAM layer were immunohistochemically stained with Ki67; (**e**) HE staining of 92.1 cells; (**f**) HE staining of 92.1 cells in the CAM layer. Abbreviation: AC = apoptotic cells, ED = chorionic ectoderm, FFPE = formalin-fixed, paraffin-embedded, MD = chorionic mesoderm, MG = Matrigel graft, NC = necrotic cells, PC = proliferating cells, VA = vascularization. Scale bars = 100 µm (**a**–**c**,**f**), 50 µm (**d**,**e**).

Different histochemical and immunohistochemical stainings were performed to characterize the human UM cells on the CAM layer. PAS staining was used to distinguish the CAM layer and the Matrigel grafts, including human 92.1 cells (Figure 3a). The Matrigel grafts were compact and surrounded by an ectodermal CAM layer. To identify not only human UM cells on the CAM ectoderm, but also tumor cell invasion into the CAM mesoderm, antihuman melanosome antibody and MelanA antibody were used (Figure 3b,c). Proliferating tumor cells were detected using Ki67 (SP6, Thermo Fisher Scientific, Rockford, IL, USA) (Figure 3d). In order to assess the viability of human and chicken cells, HE and PAS staining were performed to detect the necrosis (loss of cell definition, nuclear degradation) and apoptosis (nucleus fragmentation, chromatin condensation) of UM cells (Figure 3a,e–f). This also determined intratumoral and embryonic vascularization.

**Figure 4:**
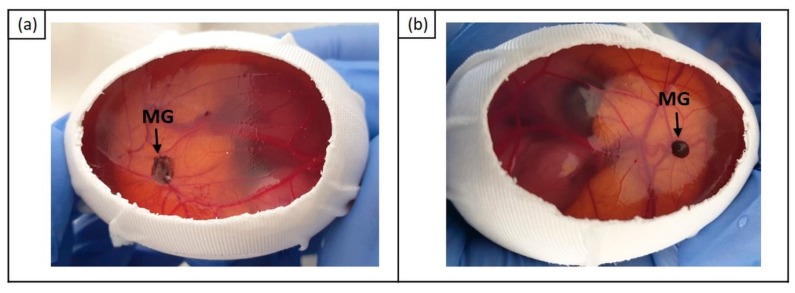
Images of chick embryo with Matrigel grafts at E16. (**a**) flat Matrigel graft; (**b**) 3D tumor organ. Abbreviation: MG = Matrigel graft.

In successfully cultivated CAMs, the cell‒Matrigel grafts were predominantly flat at E16 (Figure 4a). Approximately 10% of the cultivated Matrigel grafts successfully formed 3D tumor organs (Figure 4b).

**Figure 5:**
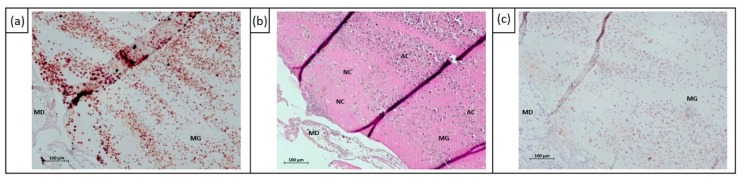
IHC stains of UM cells in the CAM at E16 following intraarterial electrochemotherapy using 2.5 µg/mL bleomycin and eight pulses of 750V/cm pulse strength. (**a**) Human UM cells 92.1 were immunohistochemically stained with antihuman melanosome marker; (**b**) the viability of cells has been controlled using PAS staining and detection of necrotic cells by hematoxylin-negative nucleus; apoptotic cells are characterized by chromatin condenses and apoptotic bodies; (**c**) Ki67 staining of CAM, including 92.1 cells, was negative. Abbreviation: AC = apoptotic cells, MD = chorionic mesoderm, MG = Matrigel graft, NC = necrotic cells. Scale bars = 100 µm.

We compared the cytotoxic effects of ECT on 3D tumor organoids at day 5 after intraarterial and intratumoral injection of bleomycin (E16). The control group consisted of cell‒Matrigel grafts without as well as after treatment with EP or chemotherapy alone. ECT with intraarterial injection of bleomycin mostly resulted in necrosis and apoptosis of tumor cells, especially in the peripheral Matrigel graft region near the chicken tissue or near blood vessels (Figure 5). Furthermore, apoptotic tumor cells have also been detected in the inner layers of the Matrigel grafts, despite the minor cytotoxic effect. Ki67+ cells were not found. Substantial differences between the application of 2.5 µg/mL and 1.0 µg/mL bleomycin have not been detected.

**Figure 6:**
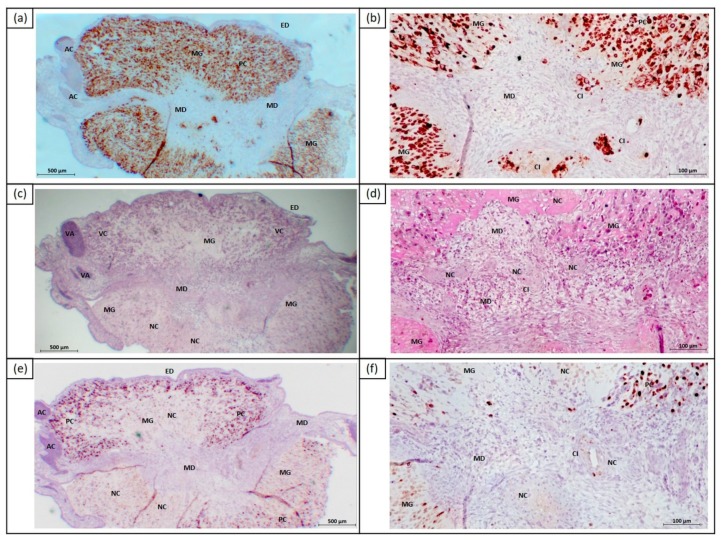
IHC stains of human 92.1 UM cells in the CAM at E16 following intratumoral ECT using 2.5µg/mL bleomycin and eight pulses 750V/cm pulse strength. (**a**) Overview of CAM including human UM cells 92.1 were immunohistochemically stained with antihuman melanosome marker (in brown); (**b**) central CAM section with surrounding Matrigel graft and central invading human 92.1 cells were immunohistochemically stained with antihuman melanosome marker (in brown); (**c**) overview of CAM including human UM cells, viability of human UM cells were controlled using hematoxylin and eosin staining (HE staining); (**d**) PAS staining of central CAM section; the majority of invading 92.1 cells were necrotic (negative for hematoxylin); (**e**) overview of CAM including human UM cells; proliferating 92.1 were immunohistochemically labeled with antihuman Ki67 marker (in brown); (**f**) Ki67 labeling of central CAM section (in brown), peripheral proliferating cells, central Ki67 negative cells. Abbreviation: AC = apoptotic cells, CI = cancer cell invasion, ED = Chorionic ectoderm, MD = chorionic mesoderm, MG = Matrigel graft, NC = necrotic cells, PC = proliferating cells, VA = vascularization, VC = vital cells. Scale bars = 500 µm (**a**,**c**,**e**), = 100 µm (**b**,**d**,**f**).

The intratumoral ECT led to large intratumoral areas of necrosis in the center of the Matrigel grafts (Figure 6). In the embryonic mesoderm, the cell death of infiltrating UM cells was observed. In peripheral regions of the Matrigel grafts, tumor cells were Ki67+ and showed almost no cytotoxic effects. Both 2.5 µg/mL and 1.0 µg/mL bleomycin caused cytotoxic effects.

**Figure 7:**
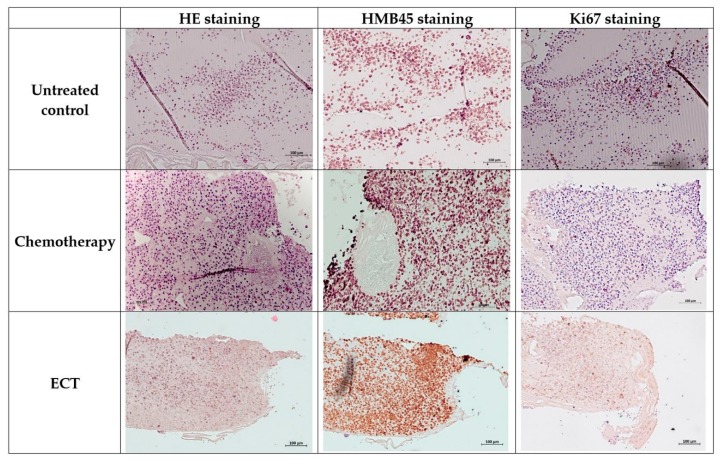
Representative IHC stains of human 92.1 UM cells in the CAM at E16 following ECT (2.5 µg/mL bleomycin and eight pulses 750 V/cm pulse strength) compared to chemotherapy alone and untreated controls. PAS staining of central CAM section with necrotic areas) and reduction of growth after ECT (negative for hematoxylin). Immunohistochemically stained with antihuman melanosome marker HMB45, large areas of necrosis after chemotherapy and after ECT with significant reduction of growth after ECT. Ki67 labeling of central CAM section, peripheral proliferating cells, central Ki67 negative cells after ECT. Scale bars = 100 µm.

Compared to ECT, in the control group samples without treatment or chemotherapy alone, no significant effect on the viability of tumor cells in Matrigel grafts could be detected (Figure 7).

## 3. Discussion

This novel study investigated the effect of ECT in vitro and in vivo, setting a milestone for further experiments and clinical application. We demonstrate the first results following ECT using lower doses than the peak plasma concentration (0.5–5.0 µg/mL) of bleomycin. Bleomycin can be administered intratumorally or intravenously [10,21,22,23]. It is a large molecule (1415.56 g/mol) that does not penetrate the intact cell membrane [22]; however, if it does, it binds to the DNA, leading to cell death [10,21,22]. This antibiotic antitumor drug is used in a wide range of malignancies [21,22]. Previous studies have proven that 500 molecules of bleomycin are required to enter the cytosol in order to cause cell death [24,25]. Pyrhonen et al. (2002) have shown the limited effect of bleomycin alone in the metastatic UM [26]. The effect of different bleomycin concentrations in four UM cells in vitro has been described in the literature [15]. This study demonstrated the positive effect of ECT in 3D spheroids. In vitro 3D culture systems simulate in vivo tumor growth characteristics and are suitable for the examination of tumor mass changes following treatment [16]. Using customized handheld electrodes, the spheroids were treated in their culture wells without disrupting the tumor environment, leading to representative results.

The response of the different UM cell lines varied following treatment. The metastatic cell lines OMM1 and MM28 were more sensitive to ECT with bleomycin. It is postulated that the cell size, shape, and membrane characteristics may affect EP [27,28]. An approximately 50% mortality rate has been reported in patients with UM as a result of hematogenous metastasis to the liver [1,2,3]. OMM1 was derived from a subcutaneous metastatic UM, while the MM28 cell line was derived from an UM liver metastasis. 92.1, Mel270, and MP46 cells were extracted from primary tumors. Mel270 showed a minor response compared to the other primary and the metastatic cell lines. These results could be associated with the origin and the characteristics of the primary tumors that gave rise to these UM cell lines. Mel270 were isolated after prior irradiation, which could explain the tumor resistance [29]. The MM28 and MP46 cells exhibit chromosome 3 loss (monosomy 3). It is known that monosomy 3 is a negative predictor of survival and metastatic disease [1,2,3,4,5,30]. Our study describes positive effects despite background chromosome 3 alterations as well as diverse cell characteristics following ECT with bleomycin. Further cell membrane characteristics were not investigated.

Hamburger and Hamilton classified in 1951 the stages of development of the chick embryo and enabled in vivo biomedical research [31]. The chick embryo is a preclinical in vivo model, which allows the study of metastatic and primary tumors due to its lack of an immune system in early development, without ethical limitations [18,19,20]. The study of tumor metastasis via the chick embryo model has been reported in a variety of cancer types including renal, colorectal, glioma prostate, and small-cell lung cancer [32,33,34,35,36,37]. Kalirai et al. confirmed that the chick embryo model is an appropriate animal model for understanding development and metastatic UM disease [19]. In the second part of our study, we examined the effect of ECT when combined with intraarterial or intratumoral administration of bleomycin. Intraarterial infusion in the proximity of the tumor was favored to avoid possible systemic adverse events of bleomycin when crossing the systemic circulation. Necrosis and apoptosis of tumor cells was documented in the peripheral graft region near vessels and near the chicken tissue. This could be attributed to a possible lack of neovascularization in the center of the tumor mass. The accumulation of the administered cytotoxic drug in the center of the tumor mass during intratumoral treatment explains the large areas of necrosis in this region. We used plate electrodes for the treatment of the UM tumors, which are suitable for the treatment of superficial lesions and, therefore, not advisable for deep-seated tumors, including UM. The use of ECT in the treatment of UM would pose a challenge to clinicians who need to access the tumor. New electrodes need to be developed in order to access the tumor without causing trauma to the sclera or to the uvea, which may facilitate metastasis. Furthermore, the extent of the thermal damage and the possible intraocular inflammatory reaction following treatment in the surrounding healthy tissue remain unknown. Complications following ECT could promptly be investigated in clinical practice. As proof of principle, one cell line of the primary tumor was used in the chick embryo model, in order to test the application and effectivity of ECT. Further experiments using other primary and metastatic UM cell lines have to be performed to evaluate the cytotoxic effect of ECT with intratumoral vs. systemic application of bleomycin.

The references in the literature to the possible use of ECT in the treatment of ocular melanoma are limited. The Collaborative Ocular Melanoma Study suggested that invasive therapeutic modalities are not associated with a lower mortality rate in comparison with eye-conserving therapies for patients with medium-sized melanomas [38]. ECT could be a less invasive therapeutic option, especially for larger nonresectable tumors, offering the advantage of avoiding the complications of chemotherapy due to the lower doses used. The application of ECT may result in a shrinkage of large tumors, which enables alternative therapy other than primary enucleation. Furthermore, ECT could allow lower drug doses for the treatment of large hepatic metastases, non-amendable for other treatment. The combination of various bleomycin concentrations as well as additional drugs with different ECT conditions requires further investigation in vitro and in vivo and poses a challenge for future research.

## 4. Materials and Methods

### 4.1. Culture of Uveal Melanoma Cell Lines

UM cell lines 92.1, Mel270, and OMM1 were kindly provided by Prof. Sarah Coupland and Dr. Helen Kalirai of the Liverpool Ocular Oncology Research Group (www.loorg.org). All three cell lines have been described as having disomy 3 and nuclear expression of the BAP1 protein. The authentication of cell lines was performed. Two further cell lines MM28 (ATCC CRL-3295) and MP46 (ATCC CRL-3298) were purchased from LGC standards in 2019. These cell lines are characterized by monosomy 3 or *BAP1* mutation [39]. Cells were cultured in complete medium containing RPMI 1640 medium with 10% FCS (92.1, Mel270, OMM1) or RPMI 1640 medium with 20% FCS (MM28, MP46), respectively. Spheroids were generated by seeding 5 × 10^3^ cells in round bottom 96 well ultra-low attachment plates (Corning, Corning, NY, USA) containing 200 µL complete medium. For the experiments three days tumor spheroids were used.

### 4.2. Treatment of Spheroids

Tumor spheroids were treated either with 200 µL complete medium containing 2.5 µg/mL bleomycin alone (chemotherapy), or with high-voltage electrical pulses electroporation (750V/8 pulses) in the absence of bleomycin (electroporation, EP) or in the presence of 2.5 µg/mL bleomycin (electrochemotherapy, ECT) using a voltage pulse generator (Cliniporator, IGEA S.p.A., Carpi, Italy). Details of the EP protocol are as follows: two parallel aluminum electrodes 4 mm apart, eight pulses, 100 µs pulse duration, 5 Hz repetition frequency, and 750 V/cm pulse strength. As a negative control, a further sample remained untreated (control). Twenty-four hours after treatment, the spheroids were washed three times with PBS and incubated in a fresh complete medium. Spheroids were harvested seven days following treatment. The analysis was performed in three to eight independent biological replicates on different dates.

### 4.3. Determination of Spheroid Growth

The growth of spheroids was analyzed seven days after treatment using bright-field microscopy by calculating the cross-sectional area of the spheroids using the image processing software program ImageJ Fiji (Dresden, Germany) [40]. The relative treatment response was calculated by comparison of the percentage of the mean cross-sectional area of the samples to the mean cross-sectional area of untreated control samples.

### 4.4. Determination of Spheroid Viability

Spheroids of UM cell lines are flat-faced spheres. Thus, MTT assay can be used as an indirect method to analyze the viability of the spheroids. MTT assay (MerckMillipore, Darmstadt, Germany) was performed seven days after treatment according to the manufacturer’s instructions. In short, the medium was removed from spheroids, and 110 µL fresh medium including 10 µL AB solution was added. After four hours, the formacan was resuspended using 100 µL isopropanol. Then 200 µL spheroid solution were transferred to a 96-well plate and spectrophotometrically analyzed using Tecan Infinity M Plex (Salzburg, Austria).

### 4.5. In Vivo Chick Embryo Chorioallantoic Membrane Assay (CAM) Assay as a Model to Investigate Tumor Growth and Viability after ECT

**Figure 8:**
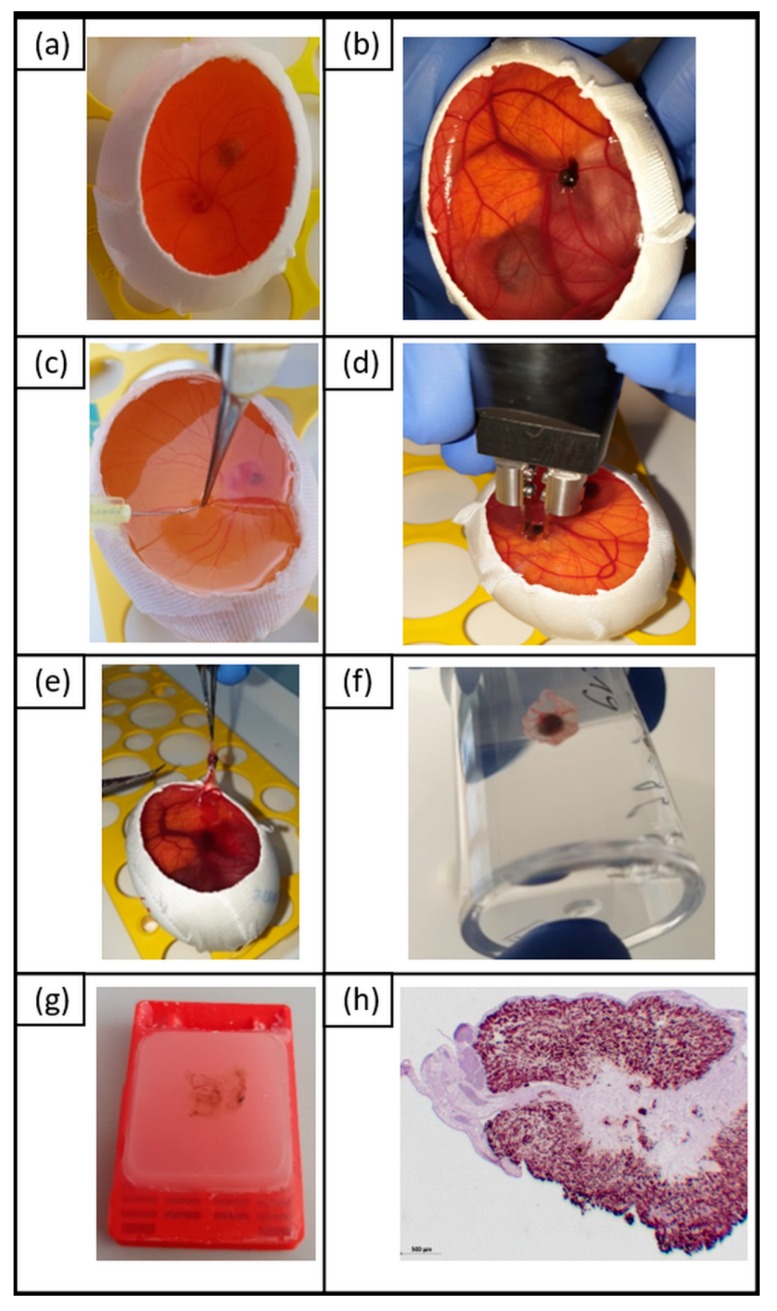
Overview of in vivo CAM assay to study the impact of ECT on human UM cells 92.1. (**a**) Chick embryo with Matrigel graft at E4; (**b**) chick embryo with Matrigel graft at E11; (**c**) intraarterial injection of bleomycin at E11; (**d**) EP of 3D tumor organoid at E11; (**e**) rejection of 3D tumor organoid at E16; (**f**) fixating cell‒Matrigel grafts with surrounding chick embryo tissue in 4% paraformaldehyde at E16; (**g**) FFPE sample of cell‒Matrigel graft with surrounding chick embryo; (**h**) immunohistochemical staining of human tumor cells in FFPE samples of CAM; tumor cells are stained with antihuman melanosome (in brown).

Fertilized chicken eggs were obtained from Ovovac GmbH, Thallwitz, Germany. Eggs were incubated in an incubator (400-RD, Bruja GmbH) at 37.7 °C with 60% humidity. At embryonic day three (E3), approximately 7‒8 mL of albumin were removed from the apical side of the egg using a 20 gauge needle and syringe without detaching the embryo or the yolk. At E4 a small window was cut in the top surface (Figure 8a). UM cell suspensions (2 × 10^6^ cells) were mixed 1:1 with Matrigel (Corning B.V.) in a total volume of 40 µL. Cell‒Matrigel grafts were placed on top of the CAM near to chicken vessels. The window was resealed with adhesive tape and eggs were incubated at 37.7 °C until E11. At E11, the samples were treated by ECT. In detail, 50 µL bleomycin (2.5 µg/mL, 1 µg/mL) were injected into a chicken artery using a 30 gauge needle (intraarterial injection) or into a tumor-formed cell‒Matrigel graft (intratumoral injection) (Figure 8b,c). Subsequently, Matrigel grafts including human UM cells were treated with high-voltage electrical pulses—known as EP—using a voltage pulse generator (Cliniporator, IGEA S.p.A., Carpi, Italy) (Figure 8d). The following EP settings were used: two parallel aluminum electrodes 4 mm apart, eight pulses, 100 µs pulse duration, 5 Hz repetition frequency, and

(A) 750 V/cm pulse strength or
(B) 1000 V/cm pulse strength.

As a control, untreated samples (control), as well as samples with bleomycin-only treatment (chemotherapy) and samples with only EP treatment, were included in the analyses. Eggs were returned to the incubator until E16. Matrigel grafts with surrounding CAM tissue were harvested from each embryo and fixed in 4% paraformaldehyde for 24 h and embedded in paraffin at E16 (Figure 8e–g). Serial sections (4 µm) were stained by periodic acid Schiff reaction (PAS staining) and hematoxylin/eosin (HE staining) to identify the Matrigel grafts on the CAM, including the human UM and viability of cells, by determining cytotoxic effects like apoptosis and necrosis as well as vascularization. Further slides were prepared for immunohistochemical staining (Figure 8h).

### 4.6. Immunohistochemistry

Paraffin sections were incubated at 60 °C for 2 h and afterwards dehydrated with xylene and decreasing ethanol concentration, followed by distilled water. Antigen retrieval was performed using 10 mM sodium citrate solution (pH6) for 30 min in a water bath at 95 °C. The endogenous peroxidase activity of the sections was blocked by using 3% H_2_O_2_ for 10 min. Antigen detection, including blocking of background staining, was performed using a ZytoChem Plus HRP Polymer Kit (Zytomed Systems GmbH, Berlin, Germany). Tissue sections were incubated with primary antibodies for 30 min at 37 °C (Ki67 monoclonal antibody 1:10 (SP6, Thermo Fisher Scientific, Rockford, IL, USA ), MelanA monoclonal antibody 1:50 (A103, Abcam, Cambridge, UK), and antihuman melanosome 1:100 (HMB45, Dako, Carpinteria, CA, USA), followed by DAB/HRP detection. Sections were counterstained with Mayer’s hematoxylin solution for 30 s.

### 4.7. Statistical Analysis

All analysis was performed using IBM SPSS statistics 25 (IBM, Armonk, NY, USA) for Windows software. Student’s *t*-test was used to determine the statistical significance of spheroid viability and growth. Results were determined to be significantly different if *p*-values were ≤ 0.05.

## 5. Conclusions

This study presents the positive results of ECT with bleomycin in metastatic as well as primary UM 3D spheroids with or without monosomy 3. Moreover, we discussed the use of the chick embryo model in UM and its treatment with ECT. The application of ECT may result in globe-sparing therapy for large UM. Additionally, the use of ECT could be considered in localized metastatic UM. Further investigations in vivo and in vivo are required in order to establish this new modality as another therapeutic option in the treatment of UM.

## Figures and Tables

**Table 1:** Average differences in spheroid growth of electroporation alone (EP), chemotherapy using bleomycin (2.5µg/mL) alone and electrochemotherapy (ECT) compared to untreated controls at day seven following treatment. Significant reduced cross-sectional areas (*p* value ≤ 0.05) are highlighted in bold letters.

Cell line	Average Differences Compared to Untreated Controls (in %)		
	EP	Chemotherapy	ECT
Primary UM cell lines			
MP46	−3.0	**19.8**	**18.3**
92.1	**6.8**	2.9	**14.4**
Mel270	−0.7	**17.5**	**25.4**
Mean differences	1.1	13.4	19.4
Metastatic UM cell lines			
MM28	**13.9**	**33.0**	**79.9**
OMM1	10.9	**14.0**	**32.6**
Mean differences	12.4	23.5	56.2

**Table 2:** Average differences in viability of electroporation alone (EP), chemotherapy alone using bleomycin and electrochemotherapy (ECT) compared to untreated controls at day seven following treatment. Viability was determined using MTT assay. Significant reduced cross-sectional areas (*p* value ≤ 0.05) are highlighted in bold.

Cell line	Average Differences Compared to Untreated Controls (in %)		
	EP	Chemotherapy	ECT
Primary UM cell lines			
MP46	−1.5	**12.8**	**49.3**
92.1	5.7	5.7	**29.4**
Mel270	4.8	−3.0	**1.9**
Mean differences	−0.2	5.2	26.8
Metastatic UM cell lines			
MM28	7.0	8.3	**58.1**
OMM1	−2.4	26.7	25.0
Mean differences	2.3	17.5	41.5
